# Complete Genome Sequence of Enterotoxigenic Escherichia coli Podophage LL11

**DOI:** 10.1128/MRA.00693-19

**Published:** 2019-08-08

**Authors:** Matthew Theodore, Lauren Lessor, Chandler O’Leary, Rohit Kongari, Jason Gill, Mei Liu

**Affiliations:** a Center for Phage Technology, Texas A&M University, College Station, Texas, USA; Loyola University Chicago

## Abstract

Enterotoxigenic Escherichia coli (ETEC) is an opportunistic pathogen that commonly causes foodborne illness. Study of bacteriophages against this pathogen could be useful to develop alternative treatment approaches. Here, we present the complete genome sequence of LL11, a T7-like podophage that infects ETEC.

## ANNOUNCEMENT

Enterotoxigenic Escherichia coli (ETEC) is a global health concern due to its ability to cause traveler’s diarrhea, as well as the increase in the emergence of antibiotic-resistant strains (1–3). The study of bacteriophages that infect ETEC provides insight into alternative therapeutic and preventative approaches (4, 5). Here, we present the complete genome sequence of podophage LL11, which infects ETEC.

Podophage LL11 was isolated using a clinical ETEC isolate from a municipal wastewater treatment plant in College Station, TX, in 2011. Host bacteria were cultured on LB broth or agar (Difco) at 37°C with aeration. The phage was cultured and propagated by the soft-agar overlay method (6). It was identified as a podophage using negative-stain transmission electron microscopy performed at the Texas A&M University Microscopy and Imaging Center as described previously (7). Phage genomic DNA was prepared using a modified Promega Wizard DNA cleanup kit protocol as described previously (7). Phage LL11 DNA was prepared using the GS FLX Titanium general DNA library preparation kit and sequenced by FLX Titanium 454 pyrosequencing at the Emory GRA Genome Center (Emory University, GA); trimmed FLX Titanium sequence reads were assembled into a single contig at 96.3-fold coverage using Newbler 2.5.3 (454 Life Sciences) with default settings. Contig completion was confirmed by PCR using primers (5′-AGCAATGCCTTGCCTAAG-3′, 5′-AGTCGTATTCGTCTGGTTAAAG-3′) facing away from the center of the assembled contig and by Sanger sequencing of the resulting product, with the contig sequence manually corrected to match the resulting Sanger sequencing read. GLIMMER 3.0 (8) and MetaGeneAnnotator 1.0 (9) were used to predict protein coding genes, with manual correction for appropriate gene starts, and tRNA genes were predicted with ARAGORN 2.36 (10). Rho-independent termination sites were identified via TransTermHP (http://transterm.cbcb.umd.edu/). Sequence similarity searches were conducted by BLASTp 2.2.28 (11) against the NCBI nonredundant (nr), UniProt Swiss-Prot (12), and TrEMBL databases. InterProScan 5.15-54.0 (13), LipoP (14), and TMHMM 2.0 (15) were used to predict protein function. All analyses were conducted at default settings via the CPT Galaxy (16) and WebApollo (17) interfaces (https://cpt.tamu.edu).

The complete genome of LL11 is 44,185 bp and is most similar to those of the phages K1-5 (GenBank accession no. NC_008152) and K1E (AM084415) (18) with 74% and 57% overall similarity at the nucleotide level, respectively, as determined by BLASTn. LL11 has a GC content of 45.1% and contains 55 protein coding genes, most of which had homologues in phage T7 (NC_001604). The annotated genes include those responsible for morphogenesis, such as the tail spike, tail protein, major capsid protein, portal protein, and scaffolding protein, genes involved in DNA replication, such as DNA polymerase, RNA polymerase, and DNA primase, lysis genes, such as a class-II holin, endolysin with a conserved lysozyme domain, and a separated spanin pair. Similarly to phage K1-5 (AY370674), LL11 also has two predicted tail fiber proteins related to tail fiber proteins of K1-5 (NC_008152), with one identical to K5 lyase (YP_654147) and the other distantly related to K1 endosialidase (YP_654148). These tail fiber proteins could allow LL11 to infect multiple host strains using different host receptor proteins in a manner similar to that of phage K1-5 (19).

### Data availability.

The genome sequence of phage LL11 was submitted to GenBank under accession no. MH729818. The associated BioProject, SRA, and BioSample accession numbers are PRJNA222858, SRR9214597, and SAMN11975578, respectively.
